# The Normativity of Law: Has the Dispositional Model Solved our Problem?^*^

**DOI:** 10.1093/ojls/gqac012

**Published:** 2022-06-23

**Authors:** Andreas Vassiliou

**Keywords:** normativity, practical reasoning, legal theory, jurisprudence, legal philosophy, Raz

## Abstract

In *Legal Directives and Practical Reasons*, Noam Gur has presented a novel account, called the dispositional model, to explain how law bears on our normative practical reasons. Gur holds that his model is superior to the current models, namely the standard weighing model and Joseph Raz’s exclusionary model. Although his work provides useful insights into the practical impact of law, I argue that: (i) his challenge against the exclusionary model is valid only insofar as one accepts Raz’s normal justification thesis and dependence thesis; (ii) his argument against the weighing model misses its target, because it attacks the model as a decision-making method, not as an account of practical reason; and (iii) his dispositional model solely constitutes a decision-making strategy and does not offer a third alternative answer to the question of how law affects our normative practical reasons. Hence, the dispositional model is not a competitor to the weighing and the exclusionary model, and the problem of accounting for the normativity of law remains.

## 1. Introduction

Let me tell you something you probably already know: most articles with a yes/no question in their title give a negative answer. This paper is no exception. In *Legal Directives and Practical Reasons*, Noam Gur has taken up the task of providing a novel model accounting for ‘the way in which law bears (or can bear) on our reasons’.^1^ There are two models with which he juxtaposes his view: the standard comparative approach, which he calls the *weighing model*, and Joseph Raz’s pre-emption thesis, or, as I will dub it, the *exclusionary model*. Gur argues that both models suffer from significant deficiencies and proposes an alternative account, which he has named the *dispositional model*. Although his work provides some useful insights into the relationship between law and reasons, I will argue that his project is ultimately unsuccessful. First, both current models can survive his attacks. Second, his own model is not really a third alternative view to the other models. Hence, the problem of accounting for the normativity of law remains.

Here is a short roadmap for this review article. *Legal Directives and Practical Reasons* is divided into three parts. The first two parts challenge the exclusionary model and the weighing model, while the third part presents the dispositional model. The article will neatly follow this order of discussion. After some brief introductory points, it will begin by elaborating why Gur’s primary attack on the exclusionary model misses the mark. What Gur shows at best is that Raz cannot maintain his pre-emption thesis (the exclusionary model in law) along with his normal justification thesis and dependence thesis. But this does not necessarily speak against the former thesis, only against maintaining all three theses. The article will then move on to explain why the main objection of Gur against the weighing (comparative) model misses the point too. This is because he attacks the model qua method of decision making, not qua theory of practical reason. Thus, his challenge leaves the model intact. The article will finally turn to the dispositional model. It will demonstrate how, in keeping on with his previous fallacy, Gur has developed a method of decision making, which he then compares with the two other models. This is a red herring, though. The two models are theories of practical reason and aim to explain how different phenomena, including law, affect what we ought to do. Conversely, the dispositional model lacks the resources to provide such an account and only addresses the question of how we should go about deciding what to do in a relatively good legal system. Therefore, it fails to offer a competing answer to the question of how law affects our reasons.

Given the nature of a review article, my main analysis will unavoidably downplay many of the virtues of Gur’s work. Drawing on empirical studies in psychology, the philosophy of disposition and sociological research on the common causes of law compliance, his book brings a refreshing approach to the current literature in legal theory and offers an outstanding example of how interdisciplinary research is to be conducted. It reveals that legal theorists have probably paid too little attention to issues concerning our attitudes towards the law, the effect of biases on our conformity with legal rules and the need to educate ourselves in ways that will allow us to better respond to legal directives. Most importantly, the book offers an invaluable guide through the labyrinthine philosophical discourse on the intricate interplay between law and practical reason. It is not an exaggeration to say that Gur has gathered most, if not all, of the relevant contemporary literature on the subject. However, the real merit of his work lies in the conciseness and simplicity with which these abstract ideas are presented and elaborated. As the title promises, *Legal Directives and Practical Reasons* does an exceptional job in illuminating the emerging project of accounting for legal phenomena in virtue of reasons. It fleshes out the competing accounts of the normativity of law and brings to the fore their explanatory power with real-life examples. But it does not stop there. It also shows the potential limits of the current discussions and explores the prospects of expanding their scope by introducing us to the idea that the existence of legal systems can have significant implications on our reasons for attitude. It is therefore an essential reading for anyone interested in understanding and working on the rationalist enterprise in law. Having said that, I must now leave these great attributes aside and discuss why, despite its virtues, Gur’s approach cannot get off the ground.

## 2. Clearing the Ground

The short roadmap above will have already highlighted the importance of maintaining clarity in our discussion. Setting our terminology straight can be a dull task. Nevertheless, it is important to disentangle some notions right away. First, what ‘reasons’ does the question ‘How does law bear (or can bear) on our reasons?’ refer to? To answer this, we need to know why this question is important for the participants in the debate. For a long time, legal theorists have been trying to account for *obligations* in law. How come, for example, the fact that during a pandemic the legislature enacts a bill stipulating that we ought to refrain from meeting with people outside our household has an impact on what actions we have an obligation to perform or refrain from?

Nowadays, this discussion is commonly made in terms of *normative reasons for action*. These reasons are species of a broader category commonly called normative practical reasons,^2^ which are considerations that count in favour of an agent performing an action or having an attitude under certain circumstances.^3^ The consideration, for instance, that my favourite band is playing in a music festival nearby tonight is (or provides) a reason counting in favour of me going to that festival tonight. Gur too acknowledges that the question at hand is about normative reasons. Yet, he suggests in his introduction that restricting our attention to normative reasons for *action* ‘would prematurely preclude the possibility that at least part of law’s potential normative significance is to be understood in terms of reasons that, although not reasons for action themselves, have indirect pertinence to our actions’.^4^ Thus, he proposes that we should understand the question as referring not only to normative reasons for action, but also to normative reasons for *attitude*. We will see later how broadening the original question in this way leads his model astray.

For now, I should illustrate another crucial distinction mentioned before between theories of practical reason and methods of decision making (or practical reasoning).^5^ A theory of practical reason addresses the questions of what kinds of practical reasons obtain, when these reasons conflict with each other, how these conflicts are resolved and, most importantly, what agents ought to do in light of their different and commonly conflicting reasons. Conversely, the question that decision-making methods answer is not what agents ought to do, but how they should reach the right decision on what they ought to do. Of course, deciding to do something is itself a mental act which might be a necessary means for doing what one ought. Thus, a decision-making model suggesting that a certain deliberative strategy is optimific under some circumstances can entail that an agent has a normative reason, and (other things being equal) ought, to structure their deliberative process or the environment within which they deliberate in a particular way. However, the subject matter of these models is exhausted in the procedure towards reaching some decision on what action an agent will perform. Such models remain silent on the question of what makes it the case that this action is the one which the agent ought to perform. It is one thing to ask how I ought to *reach a decision* on whether to go to the music festival, it is another to ask whether I ought to *go* to the festival.

On that note, we should now briefly examine the main claims of the weighing and the exclusionary model on the questions of how practical reasons conflict in general and what agents ought to do. This will allow us later to better understand their specific answers to the question of how law affects our normative practical reasons.

We will begin with the standard account of practical reason, the weighing (comparative) model.^6^ In outline, the model stipulates that: (i) practical reasons are considerations that count in favour of an agent performing an action or having an attitude under specific circumstances; (ii) reasons conflict with each other when they count in favour of incompatible actions or attitudes; (iii) these conflicts are resolved by the relative weight of the reasons compared, with the weightier reasons defeating on balance (outweighing) the less weighty ones; and (iv) one ought, all things considered, to respond to the undefeated reason on balance.

In the 1970s, Joseph Raz argued that the weighing model presents only a half-truth of the normative story and developed an alternative account of practical reason, the exclusionary model.^7^ His model holds that, in *addition* to ordinary (first-order) reasons, which compete in weight with each other when in conflict: (i) there are second-order reasons to act or to not act for (ie be motivated by) some other reasons—the second-order reasons to *not* act for some reasons are called ‘exclusionary reasons’; (ii) an exclusionary reason conflicts with a first-order reason when it requires an agent to not act for the latter reason—the first-order reason is said to be within the ‘scope’ of the exclusionary reason; (iii) in such conflicts, the exclusionary reason always defeats (excludes) the first-order reason regardless of the latter’s weight; and (iv) one ought, all things considered, to not act for reasons excluded by the undefeated exclusionary reason.^8^

Raz has argued that his model can provide a better account of rules, decisions, promises and so on.^9^ The notion of exclusionary reasons has also been used by other theorists to explain a variety of normative phenomena, ranging from respect and trust to occupancy rights and the moral standing to deflect directives.^10^ As far as legal theory is concerned, the exclusionary model contends that the authoritative directives of legal institutions give rise not only to first-order reasons, as per the weighing model, but also to exclusionary reasons. An interesting upshot of this is that the choice we make between the two models is likely to determine the position we take in the long-standing debate between positivism and anti-positivism.^11^ Let us see how.

## 3. The Exclusionary Model

Following in the footsteps of Gur, I will begin with the pre-emption thesis, ie the application of the exclusionary model in the case of law and, in partuclar, of legitimate legal authority. Raz’s seminal theory will be familiar to most of us. Nonetheless, for reasons that will become evident when we examine Gur’s challenge, I should start off by pointing out that the pre-emption thesis constitutes only part of Raz’s account of the normativity of law. In his theory, the explanatory mechanism of how law provides us with reasons for action involves two stages.^12^ In the *first stage*, the fact that a legal institution issues an authoritative directive is supposed to give us by this act of communication a systematic combination of two types of reasons, a first-order reason to perform some action and a second-order exclusionary reason to not act for the first-order reasons provided by the merits of the case; this combination of reasons constitutes a ‘pre-emptive’ reason.^13^ So, the fact that a legal institution issues a directive stipulating that we should not meet with people outside our household in times of a pandemic is supposed to give us a pre-emptive reason to do so: that is, a first-order reason to not meet with these people and an exclusionary reason excluding our first-order reasons pertaining to the merits of the case. This is the pre-emption thesis of Raz’s theory.

As you might have noticed, I have said that the enactment of an authoritative directive is only *supposed* to give us pre-emptive reasons. This is because the enactment will genuinely provide us with such reasons only if the legal institution has justified authority—viz the normative power to provide us with these reasons by the very act of communicating its intention to do so. This is determined in the *second stage* of the account. A practical authority is normally justified when it offers us a valuable service through its directives. This service lies in the fact that we will better conform to our underlying undefeated first-order reasons (ie perform the action required by them) if we do not try to comply with them directly (ie perform the action required by them *by acting for* them) but try instead to comply with the directive (ie perform the action required by the directive *by refraining from acting for* the reasons excluded by this directive). This is the normal justification thesis. Moreover, to achieve our conformity to the right reasons, the authority should base its directives on the reasons which already apply to us independently of these directives and are relevant to the merits of the case. This is the dependence thesis. Taken together, these two theses articulate ‘the service conception of the function of authorities’.^14^ So, if the legal institution generally issues directives which are based on our ‘dependent’ reasons and help us often enough to better conform with our underlying undefeated reasons of protecting our personal and public health, then its specific directive to not meet with people outside our household during the pandemic will be justified and will give us a pre-emptive reason to act accordingly.

As Raz acknowledges, an important motivation for developing his exclusionary model has been to ‘provide a foundation for a theory of law’, namely exclusive legal positivism.^15^ This theory holds that the existence and content of law is determined by social facts alone, not by moral facts. It is contrasted with other approaches in legal philosophy, such as anti-positivism or inclusive positivism, which hold that law is or can be also determined by moral facts. So, how does the exclusionary model pave the way for exclusive positivism? In a nutshell, Raz builds his answer on the idea that law has an authoritative nature: since law claims to have authority over its subjects, and having authority is having the normative power to generate pre-emptive reasons for them, it follows that law requires subjects not only to perform the actions towards which it directs them but also to disregard their underlying moral reasons.^16^ Hence, legal subjects must be able to identify what they ought to do according to law without relying on those moral reasons. Were it otherwise, the authoritative nature of law would be undermined. Therefore, exclusive positivists are right in holding that we are supposed to identify our legal obligations by looking solely at the social facts (ie what legal directives say) and without evaluating the relevant moral facts (ie without relying on the underlying moral reasons on which the legal directives are meant to be based).

Although this is not the only argument for exclusive positivism in the literature, Raz’s advocacy of positivism has been highly influential in jurisprudence. Hence, refuting his exclusionary model may well cut the ground from under the Razian positivist tradition. With this in mind, it is time to turn our focus to Gur’s challenge to this model. Gur attacked the exclusionary model for the first time in his article ‘Legal Directives in the Realm of Practical Reason: A Challenge to the Pre-Emption Thesis’.^17^ He has reiterated his attack in a more concise form in the first part of *Legal Directives and Practical Reasons*.^18^ His argument runs as follows.

We have seen that the exclusionary model stipulates that when a first-order reason is within the scope of an exclusionary reason, the first-order reason is *always* defeated (excluded) by the exclusionary reason; thus, the agent ought *always* to refrain from acting for this first-order reason. According to Gur, to reject the model we only need to find some counterexamples where an exclusionary theorist can deny neither (i) that there is a *valid* exclusionary reason^19^ nor (ii) that a certain first-order reason is *within the scope* of this exclusionary reason, yet there is a strong intuition that an agent *ought to act for* this first-order reason. In his earlier article, Gur claimed that even if he managed to show that there is ‘at least one such instance’, this should suffice to make his case against the exclusionary model.^20^ He has, in fact, presented two instances of this sort.

His first counterexample, dubbed ‘Situation 1’, involves a scenario where an agent receives an authoritative directive to commit a clearly immoral action in the extreme, such as when a soldier receives a military order to launch an artillery counterattack at an insurgent hideout where a lot of innocent civilians live. His second counterexample, dubbed ‘Situation 2’, involves a scenario where a general directive is not morally objectionable at first sight, but there arise some particular circumstances where disobeying the directive is supported by weighty moral reasons; for example, a driver might need to disobey a legal directive setting a specific speed limit on the highway because she needs to take a severely injured person to the hospital on time. In both cases, the agents ought intuitively to act for some first-order reasons, although these reasons are within the scope of a valid exclusionary reason and are thus supposedly defeated.

Gur thinks that the exclusionary theorist might try to deny this conclusion by claiming either that there is *no valid* exclusionary reason in these situations or that the first-order reason at stake is *outside the scope* of the exclusionary reason.^21^ If either objection proves to work, then the first-order reasons (for which the agents ought intuitively to act) will not be defeated. Hence, the exclusionary model will not lead to the counter-intuitive results suggested by Gur. The first part of his book (and his earlier article) constitutes precisely an extended response to these two objections. In rebutting them, Gur focuses on the *second stage* of Raz’s theory.

Starting with the objection that such an exclusionary reason would be invalid, Gur mainly scrutinises the normal justification thesis, which stipulates the conditions under which an authority can be justified and can thereby provide agents with valid exclusionary reasons. Gur elaborately argues that Raz cannot avoid the conclusion that in Situations 1 and 2 the legal authority could be justified. Thus, Raz cannot refute that an exclusionary reason in these scenarios could indeed be valid.^22^

To rebut the second objection from the scope of the exclusionary reason, Gur turns his primary attention to the dependence thesis and, more particularly, to Raz’s claim that the scope of exclusion of a directive covers the ‘dependent’ reasons on which the directive should be based. Gur argues that Raz cannot deny that one of the dependent reasons on which the authority should have based its directive in Situation 1 is the very first-order reason for which the agent ought intuitively to act, ie to minimise civilian casualties. Similarly, Raz cannot rule out the possibility that one of the dependent reasons in Situation 2 could be the consideration that dealing with some emergency might require driving above the speed limit. So, Raz cannot eliminate the possibility that an agent ought intuitively to act for a first-order reason which is within the scope of some exclusionary reason and is thereby defeated. It follows that in both situations the exclusionary model fails to account for the intuitively right normative result. This leads Gur to draw the strong conclusion that the pre-emption thesis must be rejected.^23^

There are two main problems with this form of attack. The first is that a Razian may well bite the bullet and not try to deny that in these examples there is a valid exclusionary reason defeating the first-order reasons for which the agents ought intuitively to act. She may retort that if, in the long run, it is justified to prevent soldiers from second-guessing each military target in Situation 1 (eg because this will result to the loss of less innocent civilian lives overall) or drivers from doubting traffic regulations case by case in Situation 2 (eg because this will lead to less road accidents overall), then it is justified to accept authority in both scenarios. On this assumption, in Situations 1 and 2, agents will have a valid exclusionary reason to not act for their moral reason to prevent serious harm, even if this might look repugnant to omniscient observers who know the merits of each case.^24^

Gur might insist that his counterexamples, especially Situation 1, lead Raz’s theory to highly counter-intuitive outcomes that vitiate the exclusionary model. But we do not need to follow this line of reasoning any further, for the brief overview of Gur’s argument above reveals a deeper problem for his challenge. Remember that his attack was supposed to be aiming at the pre-emption thesis, ie the *first stage* of Raz’s theory. However, his rebuttals of the potential objections rely on the *second stage* of the theory. So, his counterexamples, even if cogent, can only show that *insofar as* one is committed to the truth of the normal justification and dependence theses, then one cannot also hold that the pre-emption thesis is true. This could be a hard blow to Raz’s account *as a whole*. But the fact that the truth of the normal justification and the dependence theses is inconsistent with the truth of the pre-emption thesis does not necessarily speak against the truth of the latter. Although Raz himself might not wish to give up all his three theses, this does not mean that a proponent of the exclusionary model in law could not drop the normal justification or the dependence thesis to sustain the pre-emption thesis.

Besides, there are prominent theorists, such as Stephen Darwall and Scott Hershovitz, who have endorsed Raz’s pre-emption thesis yet have attacked the normal justification thesis (and subsequently the dependence thesis).^25^ Darwall has argued that the justification of an authority’s power to create exclusionary reasons requires a ‘second-personal’ relation of accountability which is absent from the normal justification thesis.^26^ Hershovitz has further sketched an alternative account for the justification of political authorities which lies not in the outcome of the directives (ie the subjects’ conformity to reasons), but in the specific roles embedded in social practices, the participation in which is mandatory and morally permissible.^27^

This is not to say that Gur’s attack has missed its target completely. If his argument by counterexamples is convincing, then he will have at least taken down an important candidate account for the justification of political authorities that could support the exclusionary model in the case of law—in fact, the very justificatory analysis of the theorist who presented the exclusionary model in the first place. Nonetheless, this is far from demonstrating that *no plausible justificatory account of legal authority*, which has been or might be proposed, can uphold the exclusionary model. Thus, his argumentative strategy falls short of showing that the pre-emption thesis must be rejected. The exclusionary model can live to fight another day, and so does the exclusive legal positivist view of Raz.

## 4. The Weighing Model

Let us now move to investigate how the weighing (comparative) model accounts for the normativity of law. In short, it contends that the actions of legal institutions alter certain normatively relevant facts, which in turn affect the balance of our first-order reasons for action. There are various ways this change can occur. Common examples offered in the literature involve making a solution to a coordination problem salient, creating a mutually beneficial participation scheme, reaching a decision through a democratic procedure, etc.^28^

In the pandemic scenario, for example, we have reasons to socially distance ourselves in order to protect our own health and minimise the transmission of the disease. Of course, taking some individual social-distancing measures might produce no or only insignificant results if the rest of the population does not take similar precautions. So, although each of us might have reasons to socially distance ourself, these reasons will only have little weight if few people practice social distancing. On the other hand, we also have reasons to maintain a satisfactory standard of living and meet with our family and friends, which might conflict with and even outweigh these health-related reasons. Against this background, when a legal directive is issued stipulating that we should refrain from meeting with people outside our household, this renders that solution to our coordination problem salient, for people will now know that other people know (and so on) that by acting in this way collectively we can drastically reduce the transmission rate of the disease. Thus, refraining from socialising can become a practice by which we too should abide to mitigate the pandemic. Moreover, if the social-distancing directive is generally enforced and other people participate in this mutually beneficial practice, it seems only fair that we also contribute to this scheme by socially distancing ourselves. Finally, if this directive has been reached through some democratic procedure in which we have had an equal opportunity to participate (eg by electing our legislative representatives), then arguably we additionally have a reason of democracy to follow the directive. As a result, the enactment and implementation of the directive can make it the case that our reasons to abide by this social-distancing arrangement will become weightier and will defeat on balance our reasons to meet with people outside our household.

The weighing model, in its turn, offers a framework that allows for developing a legal anti-positivist approach, which holds that law is determined by both social and moral facts. Since, in the weighing model, the underlying moral reasons of legal subjects are not excluded by legal directives, it is the balance of *all* relevant reasons (both the pre-existing ones and the ones that obtain in virtue of the actions of legal institutions) which determines what subjects ought to do. This fits nicely with the view of many anti-positivists who argue that legal obligations are the subset of the moral—all things considered—obligations which are either enforceable on demand by adjudicative institutions (in Ronald Dworkin’s late approach) or created, altered or reinforced by legal institutions (in Mark Greenberg’s approach).^29^ On this view, the identification of legal obligations is not exhausted in finding out what the directives of legal institutions say, but also requires recourse to moral principles that make the actions of these institutions morally and thereby legally relevant.^30^ This approach can therefore be sustained only by a model that does not hold that such moral considerations are excluded, namely the weighing model. This is not to say that subscribing to the weighing model entails endorsing legal anti-positivism. It does, however, mean that anti-positivists like Dworkin and Greenberg need to rely on some version of the weighing model if they are to uphold their view. Therefore, an attack on this model in the case of law could potentially undermine their anti-positivist approach as well.

Gur raises such an attack in the second part of *Legal Directives and Practical Reasons*.^31^ His main argument against the model holds that ‘insofar as the weighing model is understood as *a method of practical decision making* to be employed by individual actors’,^32^ it cannot allow legal authorities to adequately fulfil their function of giving people normative guidance towards right action. This is because studies in empirical psychology have proven that individual decision making is systematically susceptible to some biases (such as self-enhancement, self-serving, availability and myopic discounting biases) in the very contexts of human activity that legal directives typically regulate. The frequent operation of biases in these contexts entails that if we adopt a weighing approach as a mode of individual decision making in law, our assessment of the balance of reasons as affected by legal directives will be regularly distorted by these biases. As a result, we will be prone to mistaken conclusions about what we ought to do and thereby to unjustified violations of the law. Gur thus concludes that ‘the weighing model fails to provide *a framework of practical reasoning* within which legal authority could adequately fulfil its function as a normative guide to right conduct’.^33^

The discussion in section 2 should have already made clear why the argument misses the point. The problem lies in the rider quoted above: ‘insofar as the weighing model is understood as *a method of practical decision making* to be employed by individual actors’. The weighing model, however, is a model of *practicalreason* and does not necessarily suggest a specific weighing method of decision making. It basically answers the question of *what we ought to do* and, in the case of law, it holds that what legal subjects ought to do is a function of the relative weight of their reasons as these are affected by legal instituional action.^34^

Of course, a theorist advocating the weighing model of practical reason may well favour some weighing method of decision making. But endorsing the former does not commit one to endorse a simplistic version of the latter, which is what Gur attacks. Ruth Chang makes this point plain with respect to her formulation of the weighing model, which she calls ‘comparativism’:

First, comparativism is a view about practical reason but not about practical reasoning. There is a distinguished tradition of philosophers who have argued—persuasively in my view—that evaluative comparisons of alternatives or of the strengths of their corresponding reasons don’t explain how we should arrive at rational choice … [Comparativism is] a view in the philosophy of practical reason about what makes something what one has most or sufficient reason to choose.^35^

In an omitted footnote, Chang cites theorists who have shown that practical deliberation is a much more complex process than a simple procedure of balancing the weights of competing considerations.^36^ Their common ground is that an important aspect of our practical reasoning involves deliberating first about how values, norms or generic reasons make it the case that we have some particular reasons for action—before we weigh these reasons against one another if they are in conflict.^37^ A simplistic weighing method of *decision making* seems to miss this crucial prior step of practical deliberation. In contrast, this additional layer of the deliberation process can be accommodated by a weighing model of *practical reason*, which only holds that what we ought to do is ultimately determined by the balance of our reasons when these reasons conflict.

In this respect, Gur’s argument at best adds up to the list of objections against a crude deliberative method of weighing reasons. In fact, it could even constitute an objection to more complex individual decision-making strategies that go beyond this simplistic method, since any individual assessment of one’s practical reasons, however complex, could be susceptible to the distortive effect of biases. But his argument by no means constitutes an objection to the weighing model of practical reason. The model survives his attack without a scratch. Hence, anti-positivists can rest assured that the premise of their theories faces no threat from his protest.

## 5. The Dispositional Model

Having attacked the two models in the first two parts of his book, Gur is finally ready in the third part to present his own approach.^38^ In the opening chapter of this part, he states that the main contention of his dispositional model is that ‘a reasonably just and well-functioning legal system’ gives legal subjects a normative reason to adopt a ‘law-abiding attitude’: that is, an attitude whose conative component is ‘a *disposition*, a standing inclination, to comply with legal requirements’.^39^

In the following chapter, Gur opens with the following passage:

Suppose you are asked to choose a *decision-making procedure* to be prevalently used by subjects of the law in your society, under the assumption that its legal system is reasonably just and apt to serve valuable purposes. The following alternatives are given to you. (1) The Weighing Model … (2) The Coherent Pre-emption Thesis …^40^

On this basis, Gur goes on to elaborate why the dispositional model is a superior account qua an individual decision-making procedure compared to the two alternatives. Starting with the weighing model, he does not deny that legal directives can have the normative impact that the weighing model suggests. But he holds that the fact that there is a reasonably just and well-functioning legal system in place *also* gives legal subjects a reason to cultivate and adopt a disposition to comply with legal directives. This law-abiding disposition will exert a motivational force onto them to abide by the law, which is *independent* of their reasons in favour and against obeying a particular directive. The disposition is supposed to counteract the effect of biases whose operation systematically leads people to mistakenly ascribe more weight to their reasons against obeying the law, which in turn exert on them an inappropriately stronger motivational force for disobeying. Thus, the reason for adopting this law-abiding disposition lies in the fact that people will tend to abide by the law in cases where they ought to obey but would not have done so due to the distortive effect of certain biases. Gur admits that in some ‘divergence cases’, where such biases are absent and an agent correctly concludes that her reasons to disobey a legal directive slightly outweigh her reasons to obey, this law-abiding disposition might actually lead the agent to obey a directive which she ought to disobey.^41^ Still, he maintains that the dispositional model fares better overall, since he reminds us that the weighing model ‘opts for a *mode of reasoning* fully exposed and highly susceptible to precisely the same biases that made it necessary and justified to use legal forms of regulation in the first place’.^42^

Turning to the comparison with the exclusionary model, Gur denies that legal directives give people exclusionary reasons that *always* defeat their underlying reasons (on which these directives should be based) regardless of weight. The fact that there is a reasonably just and well-functioning legal system only entails that agents have a reason to adopt a law-abiding disposition whose motivational force remains *overridable* by the countervailing motivational force of the reasons that cut against the actions required by the legal directives. Unlike the weighing model, Gur acknowledges that the exclusionary model ‘provides a *mode of practical reasoning* relatively insulated from the types of situational bias mentioned above’.^43^ Yet, he argues that his model works better than the exclusionary model in cases where a legal directive requires the performance of clearly and extremely immoral actions, as in Situations 1 and 2 discussed before. For, if legal subjects endorse the exclusionary model (in conjunction with the normal justification thesis and the dependence thesis), they will abide by these immoral legal directives regardless of the weight of their conflicting moral reasons. Conversely, if they adopt the law-abiding disposition of Gur’s model, they will not obey such directives, since the motivational force of their law-abiding disposition is overridable and can be defeated by the motivational force of their weighty moral reasons for disobeying.

Gur draws the conclusion that his model is preferable to either of the two current models. Compared to the weighing model, legal subjects who have adopted the law-abiding attitude will be less prone to unjustified violations of legal directives. Compared to the exclusionary model, subjects with this attitude will be less likely to obey clearly immoral directives. Therefore, the dispositional model constitutes the optimal middle ground between the two models.^44^

The first thing to notice is that this comparison seems to have an obvious flaw. Gur attempts to show that his dispositional model is preferable to either alternative as ‘a decision-making procedure to be prevalently used by subjects of the law’.^45^ However, this seems a red herring. The weighing and the exclusionary model are competing accounts of practical reason that answer first and foremost the questions of how reasons conflict and what agents ought to do, not the question of how agents ought to deliberate in order to do what they ought. We have already seen that there are theorists like Chang, who endorse the weighing model of practical reason without accepting a simplistic weighing method of decision making. Similarly, Raz has made explicit in an exchange with Perry that his exclusionary model is basically an account of practical reason, not just a method of decision making. Using Perry’s terminology, he has ascertained that exclusionary reasons are ‘objective’ and not merely ‘subjective’, which means that they primarily determine ‘what one ought to do’ and not ‘how one ought to go about ensuring that one does what one ought to do’.^46^

Moreover, Gur too seemed to understand the exclusionary model as an account of practical reason in his original attack on Raz. Recall that in his earlier article he had claimed that to make his case against the exclusionary model it suffices to show that there is ‘at least one such instance’ where the exclusionary model stipulates that one has a valid exclusionary reason to not act for a reason that one intuitively ought to act for. Interestingly, in *Practical Reasons and Legal Directives*, where he repeats this argument, Gur has omitted this contention. Nonetheless, even in this latter version of his attack, he maintains that ‘Any number of [such] instances, however rare or unusual … should hold us back from accepting the pre-emption thesis’.^47^

His claims are certainly plausible insofar as he is attacking a model of practical reason. Since such a model is supposed to tell us *what agents ought to do*, the fact that in certain situations, however rare or unusual, this model stipulates that one ought to *not* act upon a reason for which she intuitively ought to act could undermine the plausibility of the account. But these contentions would make little sense if Gur were only attacking a decision-making model. It would be unreasonable to claim that if we see that an individual decision-making strategy fails in one or some rare instances, this should suffice for holding us back from accepting the strategy.^48^ What one would need to show for this purpose is that this strategy fails more often than some alternative decision-making strategy to lead us to better decisions overall. But this is not the way Gur has attacked the exclusionary model in his earlier article or in the first part of his book.

There is little, if any, doubt therefore that the two current models with which Gur juxtaposes his view are accounts of practical reason. So, for the comparison between the three models to have some merit, it must be the case that the dispositional model is also an account of practical reason. On its face, however, the model says nothing on the questions of how reasons conflict and what agents ought to do. Its main contention is merely that we have a reason to adopt a law-abiding attitude which will regularly help us obey the law when it is justified to do so. Of course, adopting this attitude can affect our *actions* in the sense of what *we will decide to do*, since we will be more likely to decide to abide by legal directives. But it will not affect our *reasons for action* and thereby what *we ought to do*, ie whether we ought to obey these legal directives or not. What Gur has put forward is a model of individual decision making in the case of law, not a model *alternative* to the two current accounts of practical reason.^49^ Thus, his comparison of the three models seems unwarranted.

This might, though, be too quick a dismissal of Gur’s view. After all, the answers of the weighing model and the exclusionary model to the question of what we ought to do in the face of practical conflicts have implications on how we should reason to resolve these conflicts and decide what to do. A proponent of the weighing model of practical reason may well contend that our decision-making procedure should not simply involve the balancing of competing considerations; yet it would be hard for her to deny that we have to do some sort of balancing when our reasons conflict. In the same vein, an exclusionary theorist will have to acknowledge that if we are to respond to our exclusionary reasons, then we should make sure that our excluded reasons do not affect our deliberations and thereby the motivations for our actions. Considering this, it seems only natural to evaluate the plausibility of the two models on the basis of these implications. If the weighing and exclusionary models can only yield methods of decision making that are far from the way we deliberate and decide, this would certainly speak against them. Furthermore, if some alternative model of practical reason yielded a method of decision making that better depicted our practical deliberations, then this would count in favour of endorsing this model instead. Given this close connection between models of practical reason and methods of decision making, we should therefore explore whether we can construct an account of practical reason out of Gur’s proposal.^50^ This would vindicate his comparison of the three models, as it would qualify his approach as a third contender in the race of explaining the normativity of law. To see if this is a plausible way out for Gur, we will need to get back to the question with which we started.^51^

Recall that the original question has been how law affects our normative reasons for *action*. We have seen that both the weighing model and the exclusionary model give a certain response to the question. The weighing model holds that the actions of legal institutions change the normatively relevant facts, which in turn affect the balance of the first-order reasons for action. In the pandemic example, this means that the social-distancing directive will make it the case that the reasons of legal subjects to not meet with people outside their household will become weightier than their conflicting reasons to socialise with them. The exclusionary model, on the other hand, holds that justified legal authorities generate through their directives pre-emptive reasons by the very act of communicating their intention to do so. Thus, when the social-distancing directive is issued by such an authority, legal subjects acquire both a reason to not meet with people outside their household and an exclusionary reason to not act for their reasons for meeting these people. Conversely, the dispositional model does not have the resources to provide a distinctive answer to this question. If you remain unsure, ask yourself how its main contention that legal subjects have a reason to adopt a law-abiding attitude under certain circumstances helps you answer the question of whether they ought to refrain from meeting with people outside their household when the social-distancing legal directive is issued. The answer is that it simply cannot.

Gur actually knows very well that he has no distinctive answer to offer to this question. This is why he says that his model ‘acknowledges’ the main claim of the weighing model in the case of law, ie ‘that the operation of law sometimes, or even often, brings into play … first-order reasons’.^52^ So, Gur accepts that the normative impact of law consists in affecting the balance of our first-order reasons, which thereby determine what we ought to do. The contribution of his model is that we should not balance these reasons plain and simple; rather, we should do this balancing after we have adopted a law-abiding attitude. We could even generalise his point and suggest that whenever there is some rather permanent authority that normally issues good directives, then we should be disposed towards following these directives when balancing our reasons. But this is hardly a substantively different account of practical reasoning from which we can infer an alternative model of practical reason. At best, it can be seen as offering a tweak to the weighing method of decision making, which is to be used when a rather permanent and trustworthy authority issues some directive. Although this proposal might have merit, Gur’s ambitions for his model were far greater than that. He wanted to present a third alternative theory that would account for the normativity of law. What he has shown instead is that in a relatively good legal system people should follow a more complex decision-making strategy than a crude weighing method if they are to be more likely to do what the *balance* of their reasons requires.

Some might reasonably wonder why Gur has presented his view as a third alternative model in the first place. The answer is to be found early in the introduction of his book, where he broadens the original question of how law affects our normative reasons for *action* and asks instead how law affects our normative reasons for *action and attitude*. The risk of this manoeuvre is that a theory answering this broader question might not be a competing view to the theories addressing the original question. This is the root of Gur’s troubles. The central claim of his dispositional model tackles the issue of what normative reasons for *attitude* arise in virtue of the existence of a relatively good legal system. Contrariwise, proponents of the weighing model and the exclusionary model disagree on the question of how law affects our normative reasons for *action*. As a result, Gur’s main contention is orthogonal to this disagreement.

Nonetheless, this last observation might open up an interesting possibility for Gur. Even if his model is not an account of practical reasons for *action* and does not offer an answer that *competes* with the one given by the weighing model to the original question of how law bears on our normative reasons for action, it could perhaps be understood as an account of practical reasons for *attitude*, which offers an answer *supplementary* to the answer of the weighing model to the broader question of how law bears on our normative reasons for action and attitude. That is, one could suggest that the weighing model addresses the first part of the broader question regarding reasons for action, whereas the dispositional model addresses the second part of this question regarding reasons for attitude.^53^

However, this suggestion would still be mistaken for an important reason: the weighing model is *not* exhausted to reasons for action but also applies to reasons for attitude. To briefly recap, it holds that normative practical reasons are considerations counting in favour of an agent *performing an action or having an attitude* which compete in weight when they count in favour of incompatible actions or attitudes. It is true that in the case of law weighing theorists have focused their attention on reasons for action. But this is only because this has been the crux of the debate and the issue contested by Raz’s alternative model.^54^ It does not entail that the mechanism of the weighing model does not work for reasons for attitude. As with reasons for action, the model suggests that agents ought to adopt the attitude that is supported by the balance of their reasons for attitude—some of which might also obtain in virtue of law. So, the weighing model is a complete model of practical reason that holds for both reasons for action and reasons for attitude.

The issue then becomes whether the dispositional model offers an alternative account of practical reasons for attitude that *competes* with the weighing model in regard to those reasons. Now, we might think that at least this proposal must hold water. This would be true, though, only if we could infer from Gur’s model a *distinctive* claim about how reasons for attitude conflict and how these conflicts are resolved so as to determine what attitudes legal subjects ought to adopt. But the dispositional model lacks the resources to provide such a claim too. This is no wonder. In the same way that we could not possibly construct an account of practical reasons for action out of a hypothetical claim that legal subjects have a normative reason to obey legal directives in a relatively good legal system, we cannot construct an account of practical reasons for attitude out of Gur’s bare claim that legal subjects have a normative reason to adopt a law-abiding attitude in such a legal system. Therefore, the dispositional model will have to ‘acknowledge’ the contentions of the weighing model not only about reasons for action, but also about reasons for attitude.

Here is an example to drive this point home. Imagine a reasonably just and well-functioning legal system where legislators generally enact good laws in every area of practical life. According to Gur, this gives legal subjects a reason to hold a general law-abiding attitude, whose conative component is a disposition to obey legal directives. Let us assume that during a protracted stage of emergency the legislators are failing to give proper guidance in some domain of regulation, eg by taking overly restrictive measures during a perennial pandemic. The fact that they consistently fail in this regard could plausibly give people a reason to hold a law-*disobeying* attitude towards directives pertaining to the pandemic. This attitude would then be incompatible with the general law-abiding attitude. It is thus quite possible that our reason for holding a law-abiding attitude could conflict with some reason for holding a different attitude in some sphere of practical life. The moral is that insofar as we accept that the reason for the law-abiding attitude might not be the *only* reason in the realm of normative reasons for attitude, then a conflict between our reason for this law-abiding attitude and some other reason for attitude could emerge. So, the question will eventually be: what sort of attitude ought we to hold, the law-abiding attitude or the incompatible one?

The weighing model has an answer to give. It will say that the reason for this law-abiding attitude is put to the balance along with all other reasons for attitude. If our reason for the law-abiding attitude outweighs our reason for some incompatible attitude, then we ought to withhold the law-abiding attitude. If the latter reason outweighs our reason for the law-abiding attitude, then we ought to drop this attitude, at least in this domain of our life. In contrast, the dispositional model has nothing distinctive to say on this question. Gur seems yet again in need of the weighing model if he is to explain how the reason for this law-abiding attitude could prevail and whether legal subjects ought to adopt it.^55^

All in all, Gur’s main contention that we have a reason to hold a law-abiding attitude under certain circumstances can be fully accommodated by the weighing model. Gur is free, of course, to name his view a dispositional ‘model’. But his proposal, however called, cannot constitute an account of practical reasons for action or attitude. Thus, it falls short of offering an alternative answer to the question of how law affects our normative practical reasons, even when this question is broadly construed. The competing answers to the question *remain* the ones given by the weighing model and the exclusionary model. And so does the problem of accounting for the normativity of law.

## 6. Conclusion

I must conclude by noting that there has been something peculiar about my critique of Gur’s argumentation. I have been granting Gur the plausibility of many of his claims, only to deny afterwards that they can lead to the conclusions he has drawn. This should come as no surprise really. The history of philosophical discourse is filled with examples of theorists talking past each other and thinking they disagree when they actually make claims about different topics. I have taken great pains in this review article to explain why this is also the case with Gur’s approach. Even if there seems to be no way out for Gur, however, I think there might be a way forward: this is for him to drop his effort of developing an alternative account of the normativity of law on the basis of his interdisciplinary work and instead to reconceive his approach as a launch of a different non-competing project exploring the decision-making methods in law. I take it that this is a bit far from what Gur aimed for when he embarked on this project. But sometimes retreat and regroup may be the best available strategy … decision-making-wise.

## Footnotes

1
*Legal Directives* 1.

2 See Ruth Chang, ‘Practical Reasons: The Problem of Gridlock’ in Barry Dainton and Howard Robinson (eds), *Companion to Analytical Philosophy* (Bloomsbury 2013) 474ff.

3 See indicatively TM Scanlon, *What We Owe to Each Other* (Belknap Press 1998) ch 1; Jonathan Dancy, *Practical Reality* (OUP 2000) ch 1; Derek Parfit, *On What Matters*, vol 1 (OUP 2011) ch 1.

4
*Legal Directives* 3.

5 Gur uses the terms ‘practical reasoning’ and ‘decision making’ interchangeably. Although the two notions do not have precisely the same meaning, nothing said here hinges on their difference. Following Raz, I will take ‘practical reasoning’ to mean ‘the transition (not necessarily conscious) from belief in the premises to acceptance of the putative conclusion of a practical inference’ (Joseph Raz, ‘Introduction’ in Joseph Raz (ed), *Practical Reasoning* (OUP 1978) 5).

6 For some early formulations of this account, see Kurt Baier, *The Moral Point of View: A Rational Basis of Ethics* (Cornell UP 1958) ch 4; Thomas Nagel, *The Possibility of Altruism* (Princeton UP 1970) ch 7. More recently, it has been endorsed indicatively in Parfit (n 3) ch 1; John Broome, *Rationality through Reasoning* (Wiley Blackwell 2013) ch 4; Ruth Chang, ‘Comparativism: The Grounds of Rational Choice’ in Errol Lord and Barry Maguire (eds), *Weighing Reasons* (OUP 2016).

7 Joseph Raz, *Practical Reason and Norms* (2nd edn, Princeton UP 1990); Joseph Raz, ‘Facing Up: A Reply’ (1989) 62 S Cal L Rev 1153.

8 For brevity, the qualification ‘all things considered’ will hereafter be implied in ‘ought’ statements.

9 See Raz, *Practical Reason and Norms* (n 7); Joseph Raz, ‘Reasons for Action, Decisions, and Norms’ (1975) 84 Mind 481; Joseph Raz, ‘Promises and Obligations’ in PMS Hacker and J Raz (eds), *Law, Morality, and Society: Essays in Honour of HLA Hart* (OUP 1977).

10 See respectively Tim Henning, ‘Why Be Yourself? Kantian Respect and Frankfurtian Identification’ (2011) 61 Philosophy Quarterly 725; Arnon Keren, ‘Trust and Belief: A Preemptive Reasons Account’ (2014) 191 Synthese 2593; Anna Stilz, ‘Occupancy Rights and the Wrong of Removal’ (2013) 41 Philosophy & Public Affairs 324; Ori J Herstein, ‘Understanding Standing: Permission to Deflect Reasons’ (2017) 174 Philosophical Studies 3109.

11 cf Dimitrios Kyritsis, ‘Disposition Goes a Long Way: Review of N Gur, Legal Directives and Practical Reasons’ (2021) <https://ssrn.com/abstract=3991441> accessed 17 May 2022.

12 See Joseph Raz, *The Morality of Freedom* (Clarendon Press 1986) ch 3; Joseph Raz, ‘The Problem of Authority: Revisiting the Service Conception’ (2006) 90 Minn L Rev 1003. For a concise analysis of the two-stage constitutive explanatory mechanism, see Nicos Stavropoulos, ‘Legal Interpretivism’, *The Stanford Encyclopedia of Philosophy* (Spring edn, 2021) <https://plato.stanford.edu/archives/spr2021/entries/law-interpretivist/> accessed 17 May 2022.

13 This is Raz’s original claim in Joseph Raz, *The Morality of Freedom* (n 12) 42. According to his latest approach, however, agents should not act *only* for those reasons that count *against* the action required by the directive: Raz, ‘The Problem of Authority’ (n 12) 1022.

14 Raz, *The Morality of Freedom* (n 12) 55–6.

15 Raz, *Practical Reason and Norms* (n 7) postscript fn 1.

16 Joseph Raz, ‘Authority, Law, and Morality’ in *Ethics in the Public Domain: Essays in the Morality of Law and Politics* (OUP 1994).

17 Noam Gur, ‘Legal Directives in the Realm of Practical Reason: A Challenge to the Pre-emption Thesis’ (2007) 52 Am J Juris 159.

18
*Legal Directives* chs 2–4.

19 By ‘validity’ I mean the mode of existence of normative reasons.

20 Gur (n 17) 164.

21
*Legal Directives* ch 2.

22 ibid ch 3.

23 ibid ch 4.

24 Joseph Raz, *The Authority of Law: Essays on Law and Morality* (OUP 1979) 25. Note that this means not that the soldier in Situation 1, for instance, ought to launch the artillery counterattack, but only that the soldier ought to not *act for* (ie be motivated by) his excluded reasons to refrain from launching it. If the soldier does not act for these excluded reasons but, due to some miscalculation, does not launch the counterattack, then the Razian may say that this is actually a ‘fortunate’ mistake, for it is ‘better that the excluded reasons be conformed to’; see Raz, *Practical Reason and Norms* (n 7) 185.

25 For their objections against the normal justification thesis (and the dependence thesis), see Stephen Darwall, ‘Authority and Second-Personal Reasons for Acting’ in David Sobel and Steven Wall (eds), *Reasons for Action* (CUP 2009); Scott Hershovitz, ‘The Role of Authority’ (2011) 11 Philosophers’ Imprint 1–11 <quod.lib.umich.edu/p/phimp/3521354.0011.007/1> accessed 17 May 2022. For their endorsement of exclusionary reasons, see Stephen Darwall ‘Authority and Reasons: Exclusionary and Second‐Personal’ (2010) 120 Ethics 257; Hershovitz (ibid) fn 73.

26 Darwall, ‘Authority and Reasons’ (n 25).

27 Hershovitz (n 25) 11–18.

28 I draw my analysis from Mark Greenberg, ‘The Moral Impact Theory of Law’ (2014) 123 Yale LJ 1288. See further Richard E Flathman, *The Practice of Political Authority: Authority and the Authoritative* (University of Chicago Press 1980) 109–25; Larry Alexander, ‘Law and Exclusionary Reasons’ (1990) 18 Philosophical Topics 5; Donald H Regan, ‘Authority and Value: Reflections on Raz’s Morality of Freedom’ (1989) 62 S Cal L Rev 995; Emran Mian, ‘The Curious Case of Exclusionary Reasons’ (2002) 15 Can J L Juris 99. Dworkin has also briefly endorsed the weighing model in Ronald Dworkin, ‘Thirty Years On’ (2002) 115 Harv L Rev 1655, 1672.

29 Ronald Dworkin, *Justice for Hedgehogs* (Belknap Press 2011) ch 19; Greenberg (n 28).

30 See Stavropoulos (n 12).

31
*Legal Directives* ch 6.

32 ibid 110 (emphasis added).

33 ibid 131 (emphasis added).

34 This is also the way Gur originally sets up the model in the introduction of his book, where he states that what he will call ‘the *weighing* model’ is the position in the literature that ‘legal directives … cannot exclude otherwise applicable reasons, but can at most provide us with reasons that operate (and, when in conflict with other reasons, compete with them) in terms of their *weight*’ (ibid 14–15). In the same passage, there is an extensive footnote with authors who have defended a position of this type (ibid fn 61). It is only after this point that Gur gradually reconceives the weighing model as also entailing a particular weighing method of decision making, which he goes on to criticise in the second part of his book.

35 Chang, ‘Comparativism’ (n 6) 215–16.

36 See cited authors in ibid fn 3.

37 See Elizabeth Anderson, *Value in Ethics and Economics* (HUP 1993) 59–64; Henry Richardson, *Practical Reasoning about Final Ends* (CUP 1994) 7–10.

38
*Legal Directives* chs 7–9.

39 ibid 136.

40 ibid 161 (emphasis added).

41 ibid 158–60.

42 ibid 163 (emphasis added).

43 ibid 164 (emphasis added).

44 ibid 165.

45 See the intended quote at the start of this section.

46 See Raz, ‘Facing Up’ (n 7) 1162–4 along with Stephen Perry, ‘Second-Order Reasons, Uncertainty and Legal Theory’ (1989) 62 S Cal L Rev 913, 927–31.

47
*Legal Directives* 29.

48 Besides, as we saw, Gur concedes that in some ‘divergence cases’ his own decision-making model can also lead agents astray from what they ought to do.

49 See also Nate P Adams, ‘Book Review: Gur, Noam, *Legal Directives and Practical Reasons*’ (2019) 82 MLR 1179.

50 Thanks to an anonymous OJLS referee for pressing me to clarify this argument.

51 The distinction I have drawn between practical reason and practical reasoning (or decision making) is not undisputable in the literature. With this in mind, some readers of earlier drafts have suggested that there might be a different way out for Gur from the one I will investigate in the main text. Gur could perhaps abandon the value-based externalist framework of reasons, within which he develops his model (*Legal Directives* 9–10), and endorse a constructivist account of reasons instead. In this framework, a consideration is a reason for an agent to perform some action *because* her corresponding reason judgment is true and ultimately *because* the episode of her practical reasoning is sound, in the sense of complying with the constitutive rules of practical reasoning; see Andreas Müller, *Constructing Practical Reasons* (OUP 2020). In this light, Gur’s suggested mode of practical reasoning could perhaps yield a model of practical reason. Interesting as this suggestion might seem, it cannot save the day for Gur. According to his dispositional model, what makes it the case that legal subjects have reason for adopting a law-abiding disposition and thereby that they should practically reason with this disposition is the fact that they will be more likely to obey legal directives which they are *justified* in obeying, ie which they have *prior* (undefeated) *reasons* for obeying. (If they do not have such independent reasons for obeying, eg because the legal system is not reasonably just, then it will not be the case that they should practically reason with this disposition.) So, even if we assume the truth of constructivism, the explanatory order between the agents’ reasons for obeying legal directives and their practical reasoning with a law-abiding disposition will remain the same in Gur’s scheme. That is, agents should practically reason with this disposition *because* they have reasons for obeying and not vice versa—the only difference being that the reasons for obeying will obtain in virtue of an episode of sound practical reasoning instead of some value. Thus, even in the constructivist framework, Gur’s proposal that agents should practically reason with a law-abiding disposition can only be understood as a decision-making method which helps them do what they have undefeated reason for.

52
*Legal Directives* 137 and 218.

53 In a seminar of the Edinburgh Legal Theory Research Group where a draft of this review article was presented, Gur suggested that his model adopts entirely the answer of the weighing model about reasons for action and thus offers a *complete* answer to the broader question, which is simply not distinct from the answer of the weighing model with respect to reasons for action. Be that as it may, the analysis following in the main text will show that the claim of the dispositional model regarding reasons for attitude can be accommodated by the weighing model.

54 Raz, *Practical Reason and Norms* (n 7) 15ff.

55 This can also be seen in *Legal Directives*, 136, fn 2, where Gur says that the reason for the law-abiding attitude is ‘a reason that in the final analysis—after considering reasons that speak in favour of adopting other attitudes towards the law—establishes that we should adopt a law-abiding attitude; in other words, a reason that wins against reasons for adopting other attitudes towards the law’. The explanation of how this reason wins against the competing reasons is presumably left for the weighing model to handle.

## Notes

This work was supported by the Arts and Humanities Research Council [AH/R012709/1] in association with Corpus Christi College (Cowley Scholarship); the Alexander S Onassis Public Benefit Foundation; the National and Kapodistrian University of Athens (A Gazis Trust); and the AG Leventis Foundation. I am very grateful to Nicos Stavropoulos, Noam Gur, Hafsteinn Dan Kristjansson, Conor Crummey, Martin Kelly, Felipe Jiménez and Amalia Amaya Navarro. Earlier drafts of this review article were presented at the Edinburgh Legal Theory Seminar Series and at the Cambridge Legal Theory Discussion Group; many thanks to the participants in both seminars.

